# Exploring the Therapeutic Role of Coach-Guided Videoconferencing Expressive Writing in Multiple Sclerosis: A Qualitative Study

**DOI:** 10.3390/healthcare13101104

**Published:** 2025-05-09

**Authors:** Purva Pawar, Shelly M. Xie, Angel R. Varghese, Adrian Smith, Jie Gao, Elizabeth Vander Kamp, Kimberly Kirklin, Benjamin A. Jones, William R. Meador, Hon K. Yuen

**Affiliations:** 1 School of Public Health, University of Alabama at Birmingham, 1720 2nd Ave S, Birmingham, AL 35294, USA; pawar@uab.edu; 2 UAB Arts in Medicine, University of Alabama at Birmingham, Birmingham, AL 35294, USA; smxie@uab.edu (S.M.X.); esvk63@uab.edu (E.V.K.); kkirklin@uab.edu (K.K.); 3 College of Arts and Sciences, University of Alabama at Birmingham, 1720 2nd Ave S, Birmingham, AL 35294, USA; arvarghe@uab.edu; 4 Department of Occupational Therapy, School of Health Professions, University of Alabama at Birmingham, SHPB 354, 1720 2nd Ave S, Birmingham, AL 35294, USA; adrian02@uab.edu; 5 Medical Laboratory Sciences Program, Department of Clinical and Diagnostic Science, School of Health Professions (SHP), University of Alabama at Birmingham, Birmingham, AL 35294, USA; gaoj@uab.edu; 6 Division of Neuroimmunology and Multiple Sclerosis, Department of Neurology, Heersink School of Medicine, University of Alabama at Birmingham, Birmingham, AL 35294, USAwmeador@uabmc.edu (W.R.M.)

**Keywords:** expressive writing, multiple sclerosis, emotional processing, psychological resilience, therapeutic intervention

## Abstract

**Background/Objectives**: Emotional changes significantly affect people with multiple sclerosis (PwMS), often leading to negative psychological symptoms including grief. Effective management of these symptoms can foster personal growth, increase confidence, and renew hope. While expressive writing has demonstrated psychological benefits in processing trauma and chronic conditions, its effects on PwMS remain underexplored. This study aimed to address this gap by examining the experiences of PwMS who participated in a 10-week coach-guided videoconferencing expressive writing program. **Methods**: Twenty-five adults with MS from 10 different states across the United States participated in a 10-week coach-guided videoconferencing expressive writing program. After they completed the program, participants were interviewed individually via Zoom using a structured guide. Interviews explored participants’ experiences and perceptions of the program. All interviews were audio-recorded and transcribed verbatim for analysis. Interview transcripts were analyzed using thematic analysis within a phenomenological framework. **Results**: Analysis revealed four themes reflecting therapeutic benefits received from the expressive writing program. These themes included Improved Emotional Processing, Promoting Healing; Therapeutic Coping; Radical Acceptance Through Deeper Understanding and Self-Forgiveness; and Empowerment Through Self-Discovery and Transformation. **Conclusions**: The findings highlight participants’ psychological progression from emotional processing to transformation, emphasizing the program’s role in guiding them through their experiences of grief and other emotions related to their MS. This process provided them with both a sense of empowerment and a renewed outlook on life. Results suggest that expressive writing is a promising intervention that offers PwMS an accessible tool for emotional well-being and self-discovery.

## 1. Introduction

Multiple sclerosis (MS) is a chronic, progressive neurodegenerative disease that can affect the spinal cord and is classified as a nontraumatic spinal cord disorder [1]. MS is more common in women than men (2.8:1 ratio) [2] and predominantly diagnosed in young adults aged 20–40 years [3]. The autoimmune-mediated, chronic, inflammatory demyelination throughout the central nervous system results in widespread damage affecting motor functions [4]. This limits physical mobility in approximately 75% of people with MS (PwMS). In addition, about 66% experience moderately severe upper limb dysfunction (i.e., weakness or ataxia) [5,6]. The disease’s unpredictable relapses and progression and its effect on cognitive function further complicate the ability of PwMS to adapt to and cope with their condition [7,8,9].

MS disrupts occupational, social, and emotional well-being, with symptoms extending beyond motor and cognitive dysfunction to include emotional dysregulation associated with depression, anxiety, fatigue, pain, and sleep disturbances [5,10,11]. The triad of emotional dysregulation, depression, and anxiety in PwMS erodes their sense of control, self-confidence, and motivation and leaves them feeling overwhelmed by pervasive guilt, grief, and hopelessness [12]. Many view the diagnosis as a life sentence [13].

Grief is a critical yet frequently overlooked psychological challenge in MS, arising from the progressive and often irreversible loss of bodily function [12,14]. It can stem from the recognition of lost independence, identity shifts, and the inability to engage in formerly routine social activities [12]. The cyclic nature of disease progression and relapses further exacerbates these feelings, requiring PwMS to repeatedly adjust to new limitations [15]. The emotional burden increases vulnerability to depression, suicidal ideation, impaired relationships, and diminished quality of life [16,17]. In some instances, grief may be transient, but for many, it becomes a recurrent struggle, intensifying with each relapse or progressive phase of the disease [15]. The emotional toll of adapting to these changes can feel more disabling than the disease itself, requiring PwMS to undergo difficult adjustments to embrace a new sense of self [18]. Several studies have highlighted the need to address grief and sorrow in PwMS [12,15,19,20,21,22,23]. However, few therapeutic programs have been developed to support grief processing in this population.

Emotional wellness programs have shown promise in improving mental health outcomes for PwMS [24]. Traditional therapeutic approaches, such as cognitive behavioral therapy, provide structured support for managing the emotional burden arising from an MS diagnosis [25,26] and can potentially support grief processing in PwMS [27,28,29]. However, financial limitations or difficulty finding competent counselors may limit access, and some PwMS may hesitate to utilize these resources due to social stigma, transportation issues, or mobility limitations from their health condition [30]. Our literature search did not identify any studies applying cognitive behavioral therapy to the MS population with grief or sorrow as the outcome measure. This creates a need for accessible alternative interventions that effectively address emotional distress in PwMS.

Expressive writing, a form of psychological intervention that encourages individuals to explore their thoughts and emotions through first-person narratives that address sensitive topics like grief [31], has emerged as a promising tool for reducing emotional distress and fostering psychological resilience and physical health in various populations [32,33,34]. In a landmark randomized controlled trial, university student participants who wrote about traumatic experiences reported fewer health center visits and improved immune function compared to those writing on neutral topics [35,36]. Additionally, expressive writing has been shown to improve mood in people with post-traumatic stress disorder [37,38], alleviate grief in people living with traumatic SCI [39], and instill a more resilient self-image in survivors of severe trauma [40]. Moreover, its effects extend to autoimmune disorders, where it has been linked with reduced pain, fatigue, and overall disease activity in people with rheumatoid arthritis and lupus [41].

We located only three studies on expressive writing in PwMS, and all were conducted in Iran by the same research team [42,43,44]. These studies focused on body image and sexual dysfunction in women with MS [43,44] and sexual self-concept in men with MS [42]. Results indicated that expressive writing improved body image, sexual dysfunction, psychological distress, and overall well-being (e.g., depression and negative self-image) in women with MS [43,44], but did not improve sexual self-concept in men with MS [42]. Studies have investigated the impact of expressive emotional writing in two other neurodegenerative conditions, amyotrophic lateral sclerosis (ALS) [45] and Parkinson’s disease [46]. In the study involving people with ALS, an expressive emotional writing group reported greater well-being and quality of life, particularly in psychological and existential domains, compared to a no-writing control group at 3-month follow-up [45]. The study in people with Parkinson’s disease found no significant differences in any selected psychological and physiological outcomes between the expressive emotional writing group and the neutral writing group from baseline to immediately after the program. However, the expressive emotional writing group reported a significant reduction in anxiety from baseline to post program [46].

Despite these compelling findings, research on the impact of expressive writing on PwMS remains limited. The extensive physical and psychological challenges experienced by PwMS make it crucial to investigate the potential of expressive writing as a tool for alleviating grief and emotional distress. This study aims to address this need by exploring how participation in a 10-week coach-guided videoconferencing expressive writing program, either individually or in groups, may help PwMS alleviate chronic grief and sorrow and improve their overall emotional state.

## 2. Materials and Methods

### 2.1. Research Design and Ethical Approval

The 10-week expressive writing program was delivered via Zoom to PwMS, either individually or in small groups. Following the program, participants took part in one-on-one interviews. These interview transcripts were analyzed using thematic analysis within a phenomenological framework [47]. The study received ethical approval from the Institutional Review Board at the University of Alabama at Birmingham (IRB-300005546) and was registered with ClinicalTrials.gov (NCT04721717) before participant recruitment.

### 2.2. Participants

The study included PwMS who met predefined eligibility criteria. Inclusion criteria were as follows: (1) community-dwelling adults aged 18 or older living with MS; (2) ability to communicate verbally or through writing; (3) access to a computer or smartphone with home internet; and (4) ability to understand the study’s purpose and provide informed consent. Exclusion criteria included the following: severe cognitive or sensory impairments (e.g., deafness, blindness, or language barriers) that would hinder study participation. We selected eligibility criteria based on our previous expressive writing study for people with spinal cord injury (PwSCI) [39].

Study participants were recruited primarily through MS support groups across the United States, posting study opportunities on the National MS Society website and extending invitations to patients at the outpatient MS clinic (a Comprehensive Center for MS Care designated by the National MS Society) at the study university’s hospital.

### 2.3. Procedures

Participants, after providing informed consent and completing a pre-program evaluation, participated in weekly, hour-long expressive writing sessions over 10 weeks via Zoom. The sessions were led by a professional teaching artist. We selected a 10-week duration for the expressive writing program based on the feasibility and positive psychosocial health findings in our previous expressive writing study for PwSCI [39]. In addition, a meta-analysis reported that 10 sessions was the most common number of sessions for coach-guided or enhanced expressive writing programs [48]. The program in the current study introduced participants to various forms of writing, including affirmative, poetic, and transactional. The various writing prompts touched on key aspects of each participant’s journey with MS through emotional disclosure, cognitive appraisal, and exploring future outlook. Sample prompts included, “Please write about the feelings you have around living with MS” (emotional disclosure), “Please write about your experience of living with MS using mindful expression” (cognitive appraisal), and “Please write a letter to anyone and/or anything in your life you would like to forgive to live a more peaceful life” (future outlook). The writing sessions followed a structured format to facilitate expressive writing in a supportive environment. Each session included a check-in, brief breathing exercise, 12 min writing prompt, and 7 min reflective writing exercise. Participants had the option to share their work and discuss with the coach and/or others within the group. Sessions concluded with a closing reflection, and the coach documented key observations.

The writing coach, a certified compassionate listening facilitator-in-training, was trained in health-focused writing and trauma-informed arts practices. She has led writing classes for over two decades and offered writing, storytelling, and visual arts opportunities at a local hospital for the last 6 years. Her facilitation style was based on Dr. Maria Montessori’s approach to education. The coach focused on preparing a supportive environment that allowed participants to engage with the writing program as an individual experience. She created this environment by arriving early for each session, initiating an opening ritual, responding promptly to questions and comments in Zoom chats, and being attentive to how participants were feeling each day. Mostly, she listened. When participants asked questions, she often responded by sharing relevant examples. Because expressive writing centers on thoughts and emotions, the coach bore witness when participants expressed emotional responses to their writing. She refrained from offering advice, sympathy, or pat solutions. Instead, she listened and offered her presence, without attempting to fix what the participant was feeling.

After completing the program, participants were interviewed individually via Zoom using a structured guide. Interviews explored participants’ experiences and perceptions of the program and covered five main topics: reactions to the program, challenges, usefulness and satisfaction, impacts on daily life, and suggestions for improvement. The interviewers followed a structured guide but adapted to the natural flow of conversations. Member checking was used during the interviews to ensure that participants’ responses were understood accurately. All interviews were audio-recorded with consent and transcribed verbatim for analysis. To ensure confidentiality, the participants’ names on the pre-program evaluation questionnaire and interview transcripts were replaced with numerical pseudonyms. On average, the core of each interview lasted 16 ± 9 min (ranging from 5 to 48 min). Data were collected from June 2023 through May 2024.

### 2.4. Data Analysis

Thematic analysis, grounded in a phenomenological approach, was used to extract themes from interview transcripts. The interviewers and coder bracketed their biases and presuppositions related to the study topic to ensure an open approach to data collection and analysis [49,50]. The interviewers and coders had no prior clinical knowledge of MS, had no experience with expressive writing in PwMS, and had not reviewed the related literature before conducting interviews or coding.

The qualitative analysis was performed according to Braun and Clarke’s (2006) approach [51]. Transcripts were read multiple times by the first author (i.e., coder) to develop initial open codes while maintaining alignment with participants’ original meanings. Related codes were grouped into themes and subthemes through axial coding. Through intuiting, the coder engaged deeply with participants’ perspectives to interpret the meaning behind their experiences. The interpretations were consistently cross-checked against transcripts to maintain accuracy. The themes were iteratively reviewed to ensure they accurately reflected the data, and background participant information and feedback from the coach were incorporated where relevant. Finally, themes and subthemes were named and supported with participant quotes to provide context. Data analysis was conducted over a full semester.

## 3. Results

A total of 25 adults with MS completed all 10 sessions of the expressive writing program and the post-program interview; their characteristics are reported in Table 1. Participants came from 10 different US states. Most were female [n(%) = 20 (80%)], White [n(%) = 18 (72%)], married [n(%) = 17 (68%), living with someone [n(%) = 22 (88%)], had completed a bachelor’s degree or beyond [n(%) = 22 (88%)], and self-reported relapsing–remitting MS [n(%) = 17 (68%)]. The mean ± standard deviation age of participants was 50 ± 13 years (range, 35–78 years). The mean ± standard deviation duration of MS diagnosis was 12 ± 8.6 years (range, 1.5–33 years). Seventeen (68%) participants had individual sessions with the coach, while the rest had group sessions (two or three participants plus the coach).

**Figure 1:**
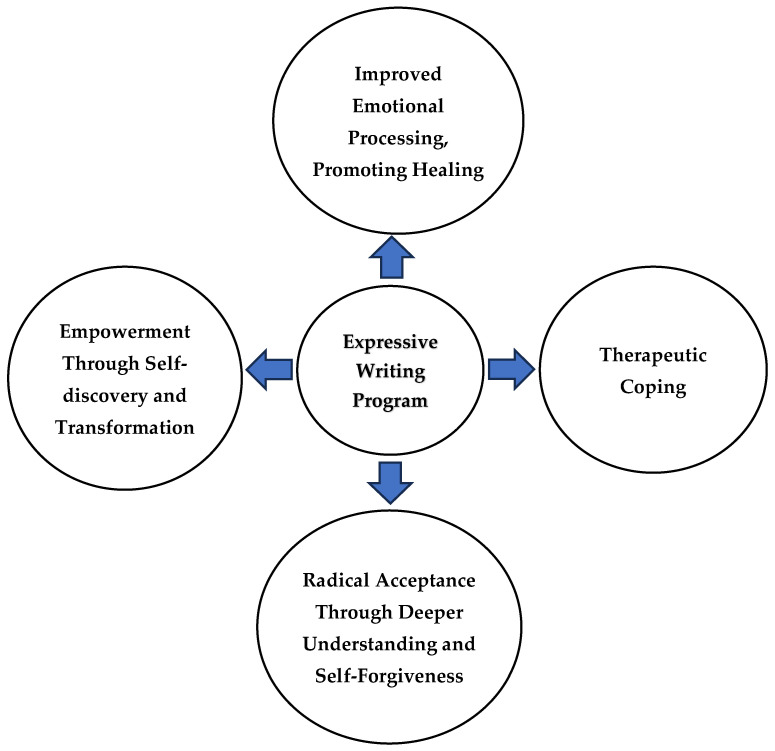
Emergent themes from participants’ experiences in a 10-week, coach-guided expressive writing program delivered via videoconferencing.

Qualitative analysis of participant experiences revealed four overarching themes and six subthemes (see Figure 1).

### 3.1. Theme 1: Improved Emotional Processing, Promoting Healing

This theme has three subthemes: (1a) Brought Forth Deep, Allowed for Reflection, (1b) Learning to Let Go, and (1c) Reclaiming Agency.

#### 3.1.1. Subtheme 1a: Brought Forth Deep, Allowed for Reflection

The program prompted participants to reflect on and process unexamined emotions. Many found that expressive writing encouraged deep introspection, uncovering feelings that they had not yet explored or expressed. This process revealed unacknowledged emotional aspects of participants’ journey with MS, helping them gain new insights and process complex emotions, which promoted personal healing and emotional growth. Participant comments related to this subtheme included the following:

*“It allowed me to kind of do a lot of self-introspection, and really kind of review my deeper feelings that I may not necessarily voice or really think about.”*

(P21)

*“They were a few moments like that where it was just like boy, didn’t know that was still in there, I think I need to work on that.”*

(P13)

#### 3.1.2. Subtheme 1b: Learning to Let Go

The unstructured nature of the writing process provided participants with the freedom to release their emotions without feeling constrained by expectations. Many found that this flexibility allowed them to let go of internal barriers and write purely for themselves, without concern for how their words might be perceived. This sense of permission facilitated a deeper emotional release and helped participants process complex emotions, allowing them to engage more freely in the healing process and find solace in the simplicity of writing for their emotional well-being. Participant comments related to this subtheme included the following:

*“Unstructured way of writing helped me to just kind of let go and I just felt like it kind of gave me permission to just do this for me.”*

(P3)

#### 3.1.3. Subtheme 1c: Reclaiming Agency

The structured, predictable nature of the program gave participants a sense of control over their emotional journey, despite ongoing challenges. The expressive writing program encouraged participants to focus on themselves and use “I”, fostering empowerment by allowing them to engage in the process on their own terms. Many found that this routine helped them regain a sense of agency, particularly in dealing with an unpredictable condition like MS. Giving participants the ability to choose when and what to share gave them more control of their emotional healing. Participant comments related to this subtheme included the following:

*“There was an empowering element to it. Where it allowed me to feel like I had a little bit more control over my uncontrollable situation.”*

(P12)

### 3.2. Theme 2: Therapeutic Coping

The writing process served as a powerful tool for helping participants cope with the emotional challenges of living with MS. Many expressed that the program helped them make sense of their condition and develop a healthier relationship with it. Writing provided an outlet for processing past struggles and current difficulties, encouraging individuals to reflect on their experiences and find peace in the process. For some, writing was not only a way to heal but also a means to overcome feelings of failure and uncertainty, helping them manage the emotional weight of their diagnosis. Overall, participants found that engaging in the program’s therapeutic writing process contributed to their emotional well-being, offering them a sense of peace and empowerment as they navigated their illness. Participant comments related to this subtheme included the following:

*“I think that I am still making sense of what multiple sclerosis means to me and I’m grateful that this has helped define or allowed me to tap into different tools to wrap my mind around it a bit more and have a healthier relationship to it.”*

(P14)

*“Writing helped to overcome my past failures, especially to deal with the issues that I was going through currently, and things that I’ve experienced in his past seven years of a diagnosis.”*

(P24)

### 3.3. Theme 3: Radical Acceptance Through Deeper Understanding and Self-Forgiveness

The writing process encouraged radical acceptance, which in turn fostered a profound sense of self-acceptance and understanding among participants, helping them confront and process their emotions with greater self-compassion. Through self-expression, they developed a greater willingness to face their feelings, moving beyond tendencies to dismiss or avoid difficult emotions. Writing provided an opportunity to release long-held emotional burdens, with some participants describing it as a vehicle for letting go of trauma that had been affecting them for years. This increased self-awareness and emotional release contributed to a sense of forgiveness, allowing individuals to accept their flaws and embrace their experiences. Participant comments related to this subtheme included the following:

*“I feel a lot more like grounded and defined in my acceptance of my disability in ways that maybe I wasn’t. I’m a little more gentle with myself. And I think I’m just like, in a little more even-keeled headspace than I was before the program started.”*

(P11)

*“I’m more kind of willing to face how I’m feeling and thinking, whereas before, I would just, oh, well, you know, hey, I’ve already dealt with that. Let’s just move on, even though I haven’t. I’ve accepted my reality more. I’m more forgiving to myself.”*

(P18)

### 3.4. Theme 4: Empowerment Through Self-Discovery and Transformation

This theme has three subthemes: (4a) Uncovered Hidden Creativity and Self-Expression, (4b) Offered a New Perspective on Life after Diagnosis, and (4c) Increased Optimism and Boosted Confidence.

#### 3.4.1. Subtheme 4a: Uncovered Hidden Creativity and Self-Expression

The program facilitated a rediscovery of creativity for many participants, allowing them to tap into aspects of themselves that they had previously neglected. For some, the experience of writing helped them reconnect with their creativity. Participants expressed that the process helped them realize they could engage in creative expression in new ways, with several noting increased motivation to explore and embrace their creativity. Through the freedom and structure provided by the writing exercises, individuals uncovered talents and perspectives that they had not recognized before, transforming their relationship with self-expression. This renewed creativity became an empowering force, giving participants a sense of fulfillment and encouraging them to continue utilizing creative outlets in their lives. Participant comments related to this subtheme included the following:

*“I realized I’m able to tap into different creative sides. I didn’t really exist.”*

(P22)

#### 3.4.2. Subtheme 4b: Offered a New Perspective on Life After Diagnosis

The program provided participants with a valuable opportunity to gain a fresh perspective on their lives and experiences following their MS diagnoses. Through guided reflection and shared experiences, participants were able to view their condition from new angles, allowing them to understand their journey more deeply. Many noted that engaging with the writing prompts and listening to others’ stories helped them develop a broader understanding of their challenges and the complexities of living with MS. This shift in perspective led to greater acceptance and a more positive outlook on their daily lives, as individuals began to see their experiences through a more nuanced, compassionate lens. Participant comments related to this subtheme included the following:

*“It’s a completely different take on how I would’ve thought of MS. And the difficulties you know that occur with it.”*

(P5)

#### 3.4.3. Subtheme 4c: Increased Optimism and Boosted Confidence

The program had a profound impact on participants’ emotional well-being, fostering increased optimism and self-confidence. Through the reflective writing process, many individuals reported feeling more positive and hopeful about their lives and their journey with MS. By giving them an outlet to process emotions and reflect on their experiences, the program empowered participants to feel better about themselves and their lives. Many noted that each session left them feeling emotionally uplifted, which contributed significantly to their sense of self-worth and confidence. Participant comments related to this subtheme included the following:

*“I feel proud of myself and definitely boosted, yeah, emotionally and personally.”*

(P27)

*“I think that it helped me become more positive. I’m, I’m more optimistic and hopeful now than I was, the writing helped. It was a very positive experience.”*

(P17)

## 4. Discussion

MS is a chronic, progressive neurodegenerative disorder that leads to a wide range of symptoms and significantly affects quality of life [17,52,53]. To the best of our knowledge, this is the first study to evaluate the impact of a coach-guided videoconferencing expressive writing program on PwMS. Our findings demonstrate that the program may serve as a viable intervention to help PwMS adopt a more positive perspective and attitude toward life. The identified themes reveal emotional and psychological progression, beginning with a supportive environment and advancing through emotional processing, coping, acceptance, and transformation. Expressive writing not only provides emotional relief but also empowers individuals with chronic conditions by fostering resilience, a sense of personal agency, and self-discovery. The program’s flexibility and creativity offer an alternative to traditional therapies, providing a promising option for improving emotional well-being and enhancing quality of life for PwMS.

The four emergent themes reflect a stage-wise progression in participants’ grief journey, from improved emotional processing and healing to therapeutic coping, radical acceptance, and empowerment through self-discovery and transformation. However, we cannot confirm these effects or isolate key points in participants’ progress through each stage of perception (i.e., themes) due to limitations in the study design. Specifically, only one interview was conducted at the conclusion of the 10-week expressive writing program, rather than after each weekly session.

A novel aspect of this study was its facilitative approach (i.e., coach-guided) to expressive writing, which allowed participants to engage in deep personal reflection and emotional processing in a nonjudgmental, supportive environment. The varied, open-ended prompts and coach guidance encouraged participants to explore different writing formats, including poetry and letters. This flexibility enhanced participants’ ability to express their emotions, aligning with previous research demonstrating the therapeutic benefits of coach-led expressive writing programs in facilitating creativity, emotional release, and meaning-making [39,54,55,56]. Notably, this approach provided participants with a sense of agency over their healing process, contributing to their emotional transformation, redefined self-identity, and development of coping strategies for future adversities.

However, this coach-guided approach is significantly different from the guided written disclosure of traumatic or stressful experiences protocol developed by Pennebaker [35], which typically involves a program of three expressive writing sessions that do not involve interaction with other individuals. None of the prior expressive writing programs for neurodegenerative populations were guided by coaches. Since this study adopted a one-group pretest–posttest design, the impact of the coach-guided component on shaping participant experience cannot be isolated and evaluated. The ability to thoroughly examine the impact of the coach in the present expressive writing program is limited without redesigning the study to incorporate a comparison group involving an expressive writing intervention without guidance from a coach. Future studies should evaluate the cost-effectiveness of individual writing sessions with and without guidance from a writing coach.

Findings of this study are consistent with the existing literature on the therapeutic benefits of expressive writing. Similarly to previous studies with other chronic illness populations, such as traumatic SCI and cancer, our study demonstrated that expressive writing facilitated self-acceptance, enhanced coping mechanisms, provided participants with a renewed perspective on life, and fostered self-forgiveness [39,45,57,58,59,60]. These shared themes across various studies further support the potential of expressive writing as an effective intervention for individuals managing chronic illnesses and emotional distress. As reported, with effective resources, PwMS can expand their emotional resilience and personal growth and gain a deeper appreciation for life, increased confidence, and renewed hope [23,61]. Recognizing and addressing the emotional impact of MS is therefore essential to improving overall well-being and supporting PwMS in navigating the emotional complexities of their condition.

This study sample is considered representative of the MS population in the United States. Among participants, 80% were women, 72% were White, and the median age was 48 years. Nationally, about 75% of people with MS are female [2,62] and 77% are White [62]. The largest proportion of the MS population is aged 45 to 54 years [62], which is consistent with the median age in this sample. Several studies have noted that women with MS were more likely to seek social support or attend a support group than men with MS [63,64] and that there are gender differences in how PwMS adjust to and cope with the condition [64,65,66]. Specifically, compared to men with MS, women with the disease were more likely to accept the condition, have a stronger belief in their ability to control it (i.e., self-efficacy) [66], and adopt coping strategies that can help them live more productively [64]. Therefore, the findings of this study are more likely to apply only to women with MS. Future studies should enroll more men with MS to evaluate whether the expressive writing program affects them differently than women.

### Strengths, Limitations, and Future Scope for Research

This study is the first to evaluate the effectiveness of expressive writing in individuals with MS, contributing to the growing body of knowledge supporting its psychological benefits. The findings are preliminary but promising, offering a foundation for future research aimed at expanding the application of coach-guided videoconferencing expressive writing to other populations and contexts.

However, several limitations must be considered when interpreting the results. First, integrating objective measures, such as healthcare utilization metrics (e.g., emergency department visits) or clinical indicators, would strengthen the credibility of future studies. Clinical indicators could include biochemical and functional parameters such as cortisol levels and heart rate variability as objective markers for stress. Additionally, psychological outcomes were only assessed immediately after the program, leaving questions about the long-term benefits of expressive writing unanswered. For instance, some of the psychological benefits, such as coping and empowerment, may not develop fully or persist, depending on whether participants maintain regular expressive writing practice and on the progression of their disease. Therefore, future research should employ longitudinal designs with follow-up assessments, including qualitative interviews at multiple time points (e.g., several months to a year post intervention) to better understand the long-term impact of the online coach-guided expressive writing program on PwMS. Moreover, investigating whether expressive writing leads to broader behavioral or physiological changes by including quantitative and objective indicators such as biomarkers could strengthen the findings and interpretations and provide further insight into the program’s impact. Another limitation is the potential bias introduced by the coach–participant dynamic (i.e., the therapeutic relationship between the writing coach and participants), which may have shaped participants’ perceptions of the expressive writing program. Finally, this study had a disproportionally high number of female participants (80%), limiting the generalizability of findings to men with MS. By addressing these limitations and building on the current findings, future research could establish expressive writing as a robust, evidence-based intervention for individuals with MS and other chronic conditions.

## 5. Conclusions

In conclusion, this study provides valuable insights into the potential of expressive writing to foster emotional resilience and psychological growth in individuals with MS. With continued research, coach-guided expressive writing could become a widely accessible and effective intervention for enhancing emotional well-being and quality of life for those coping with chronic illnesses.

## Figures and Tables

**Table 1:** Participant demographic characteristics (n = 25).

ID	Age (Years)	Gender	Race	Group or Solo Session	Marital Status	Education Level	Employment	Living Situation	Diagnosis	Duration of Diagnosis (Years)
1	43	F	B	G	M	PG	FT	WS	1	16
2	47	F	W	S	M	PG	FT	WS	1	14
3	37	F	W	G	M	PG	FT	WS	1	6
5	65	F	W	S	M	PG	H	WS	2	5
6	38	F	W	S	M	BA/BS	FT	WS	1	25
7	56	F	W	G	M	PG	H	WS	3	4
8	59	F	W	G	D	BA/BS	H	WS	2	33
9	58	M	W	G	M	PG	U	A	1	9
10	36	F	W	S	M	PG	H	WS	1	8
11	39	F	W	S	M	BA/BS	U	WS	1	2
12	37	F	W	G	M	BA/BS	H	WS	1	5
13	49	F	W	S	M	BA/BS	RT	WS	1	1.5
14	39	F	W	G	NM	BA/BS	FT	WS	1	19
15	77	M	O	G	W	PG	H	WS	1	5
16	52	F	W	S	M	PG	RT	WS	1	25
17	60	F	W	S	M	PG	U	WS	1	11
18	78	F	B	S	W	PG	U	WS	1	11
19	55	F	W	S	M	BA/BS	H	WS	3	9
20	48	M	W	S	D	BA/BS	U	A	1	13
21	73	M	W	S	M	PG	U	WS	1	10
22	39	F	B	S	M	SC	RT	WS	3	10
23	42	M	B	S	S	HS	U	WS	3	7
24	60	F	W	S	NM	SC	H	WS	3	3
26	37	F	B	S	NM	PG	PT	A	2	30
27	35	F	A	S	M	BA/BS	PT	WS	1	17

Note: Gender: F = female; M = male. Race: A = Asian; B = Black; W = White; O = other. Group or solo: G = group; S = solo. Marital status: D = divorced; M = married; NM = never married; S = separated; W = widowed. Education: HS = high school education; SC = some college education; BA/BS = bachelor’s degree; PG = post-graduate education. Employment status: FT = full-time job; PT = part-time job; H = homemaker; U = unemployed; RT = retired. Living situation: A = alone; WS = with someone. Diagnosis: 1 = relapsing–remitting MS; 2 = secondary progressive MS; 3 = primary progressive MS.

## Data Availability

The data presented in this study are available on request from the corresponding author.
